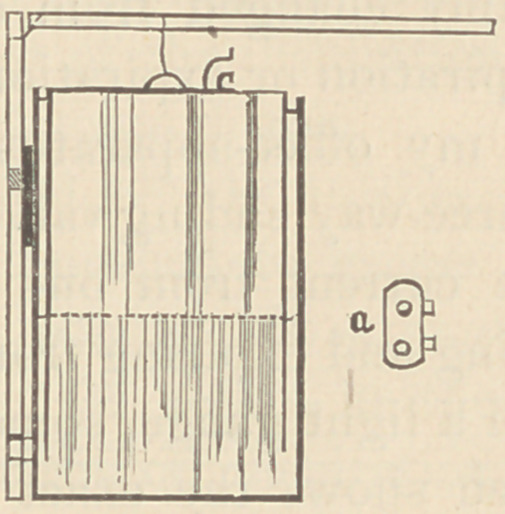# The Respiration of Compressed and Rarefied Air in Pulmonary Diseases

**Published:** 1877-10

**Authors:** F. H. Davis

**Affiliations:** Chicago


### EXAMINER.

Vol. XXXV.—OCTOBER, 1877.—No. 4.

### Original Communications.

### THE RESPIRATION OF COMPRESSED AND RAREFIED AIR IN PULMONARY DISEASES.

By F. H. Davis, M.D., Chicago.

*(A Paper Read before the Chicago Society of Physicians and Surgeons, April, 1877.)*

The application of compressed and rarefied air to the treatment of certain pulmonary and cardiac diseases, has been the subject of considerable study and experimentation during the past few years, especially in Germany.

The results of these observations and experiments have been given to the profession through the writings of Waldenberg, Fränkel and others, and in the original or through translations have attracted some attention in this country. Cohen, in the new edition of his treatise on "Inhalations," devotes a chapter to this subject, giving a very good summary of the effects to be derived from the inhalation of compressed and rarefied air, illustrated by the various apparatus devised for its practice, etc.

For stationary and office use the apparatus of Waldenberg has been the most efficient and convenient. It consists of an ordinary gasometer of sufficient size so that from 40 to 60 breaths would be required to empty it. The size, however, is immaterial, except as a matter of convenience. By raising the gasometer full of air and placing weights upon it, any desired pressure can be obtained; or attaching the weights upon the cords running over the pulleys, when the tank is depressed and emptied of air, the suction power or rarefied air is obtained. The addition of a manometer or gage to record the amount of pressure or suction obtained, is useful only for the purpose of scientific experimentations and record.

The lungs are very sensitive to pressure or suction, and will tolerate an increase or diminution of pressure of not more than from $\frac{1}{60}$ or $\frac{1}{70}$ to $\frac{1}{30}$ or $\frac{1}{40}$ of an atmosphere. The feelings of the patient are a sufficiently safe guide however, and he will naturally withdraw the tube from the mouth if the pressure becomes too great.

One thing has been lacking in order to popularize this mode of treatment and render it practically valuable. This has been a cheap, transportable apparatus, capable of being easily extemporized or manufactured whenever and wherever wanted. Patients must in many instances be enabled to follow out the treatment at their own houses where they can use it for a short time several times through the day.

One form of apparatus, Fränkel's, partially answers this purpose, in that it is not very expensive and is transportable. This is in the form of an accordeon-bellows, and held in the patient's lap, is worked by the hands. The mode of using it, however, requires considerable manual power, and is consequently tiring to the patient. The bellows are not durable, and if used for a number of different patients are liable to become contaminated by the absorption of the exhaled matters of the breath by the leather. A metal apparatus, as being readily washed and cleansed and also more durable, is preferable.

After some experimenting, I have devised the instrument shown in the accompanying cut to meet the requirements of many patients, and have now several of them in use. It consists essentially of a small metal gasometer, 15 in. in diameter by 24 in. in height. It is best made of galvanized iron, which will not rust. Two bands are soldered on to the side of the tank, into which a piece of wood is slid, reaching 12 inches above the top of the tank. To this, as a fulcrum, is hinged a wooden lever, two feet long, attached to a handle in the middle of the inner tank. A very slight exertion on the part of the patient, raising or lowering this lever, produces all the pressure or suction necessary.

This apparatus can be made by any tin-smith, and with three feet of three-quarter in. rubber-hose, costs from four to five dollars. In this, as in all the other forms of apparatus that have been devised, the patient inspires the compressed air, and expires into the ordinary atmosphere; or, *vice versa*, expires into rarified air, and inspires again the ordinary atmosphere.

Believing that, in many instances, still more marked results could be obtained by combining the two acts of inspiration of compressed air and expiration into rarified air, I had made for use in my office an apparatus, consisting of two small gasometers, as just described, placed side by side, and so arranged that one lever would raise or depress the two at once. Tubes from each gasometer pass to a double mouth-piece, so that as the lever is raised the patient expires into the one, the other being at the same time raised full of fresh air; he then inspires this fresh air as the lever is depressed, the foul air at the same time emptying itself from the first tank. Using two separate tanks, the one always to expire into, the other to inspire from, there is no possible danger of contamination of the water or walls of the gasometer used for inhalation. The simplest form of mouth-piece for this double apparatus is that shown in *figure a.* Two tin tubes are connected by a tin plate, four inches by two and one-half. This tin plate should be covered with a cloth or leather pad, which coming under the nose will prevent the current of air from passing that way. The mouth is simply changed from one tube to the other with the act of inspiration or expiration.

I have rendered my office-apparatus now quite perfect by the addition of a three-way sliding valve attached to the lever, which changes the current from one tank to the other, the patient thus inspiring and expiring from the one mouth-piece. I have also attached a light gauge, somewhat after the form of a steam-gauge, which shows the exact amount of pressure or suction used.

As thus completed, the apparatus would cost to have manufactured from fifteen to twenty dollars.

These apparatuses claim nothing new in principle, but are offered merely as suggestions to physicians who may have suitable cases for which they would like to extemporize an apparatus, or who wish to have a cheap apparatus for trial and use in their offices. The same apparatus will answer an excellent purpose for artificial respiration in cases of asphyxia, or in vivisections and physiological experiments upon animals.

In regard to the mode of action of this compressed or rarified air upon the respiration or circulation, I would quote the following epitomization from the chapter before alluded to in Cohen on Inhalation.

Inspiration of compressed air increases the pressure on the lungs, and thus augments the vital capacity ; the chest becoming expanded to a greater extent than can be accomplished by the most powerful voluntary inspiration of normal air. As a matter of course, there is a consequent pressure exerted on all the organs in contact with the lungs, and on all the contents of the thorax. The action of the heart is increased, and the pressure in the arteries augmented, so that the arterial walls are distended, and the pulse becomes full and hard. The afflux of venous blood to the right auricle is impossible, and the blood accumulates in the arterial system, the pulse becoming lowered in frequence from four to ten beats in the minute.

Expiration into compressed air diminishes the quantity of expired air in proportion to the density of the compressed air. The interchange of gases is impeded, and if pushed to excess, it will induce apnœa. It will strengthen the power of the auxiliary muscles of expiration. The effects on the circulation are similar to that of the inspiration of compressed air, but in a greater proportionate degree.

Inspiration of rarefied air diminishes the actual amount of air inspired, and if pushed to excess renders inspiration difficult, and produces apnœa. It strengthens the auxiliary muscles of inspiration. Its action in the circulation is just the opposite of the effect of inspiration of condensed air. The intrathoracic pressure is diminished, and its physical influence is exerted upon all the organs within the thorax. The heart's action is improved and marked, and the pressure in the arterial system diminishes; the pulse becomes soft, thin, compressible, and more frequent. The afflux of venous blood to the right auricle is facilitated, and blood accumulates in the pulmonary circulation, and diminishes in the general circulation. Pushed to excess it may induce hæmoptysis. Expiration into rarefied air increases the amount of air expelled from the lungs, and the lung contracts to a greater degree than it does under the most powerful effort of normal expiration. A greater proportion of carbonic acid gas is therefore given off, and the succeeding inspiration is the more powerful and effective. It thus tends to decrease the volume of emphysematous lungs, and to increase the vital capacity of the lungs. The respiratory power is increased in both its acts. It affects the circulatory apparatus similarly, to the inspiration of rarefied air, but in a much less degree. The pressure is diminished in the arterial system, and the pulse becomes soft, compressible, small and more frequent. Blood accumulates in the intrathoracic organs, and diminishes in the remaining portions of the body.

My own experience in the application of this form of treatment covers but a brief period and a limited number of cases. These cases may be grouped and the results summarized as follows:

First, a class of young persons, varying in age from twelve to twenty, of slight physique and slim build. Chests flat and narrow, and generally a tendency to a stooping of the shoulders, and a deficient expansion of the chest in respiration; also a natural disinclination to active out-door exercise and sports. This assembly of symptoms, accompanied by a sensitive condition of the air passages, caused frequent attacks of laryngeal and bronchial catarrh. Physical examination, however, revealed no evidence of tubercular deposits. In about one-half of these cases, however, the family history revealed tubercular or scrofulous taint.

The inhalation of compressed air for from five to ten minutes, once or twice a day, produced marked and rapid improvement in all these cases.

The size of the chest, on full inspiration was increased from one-half inch to one inch in the first month, and a habit of fuller, deeper breathing and a more erect carriage was established. Under the use, at the same time, of some efficient nutrient tonic, as malt extract, in combination with the hypophosphites or cod-liver oil, the general nutrition and flesh was improved, and the sensitiveness to cold and catarrhal attacks was diminished.

A second group of cases comprise those diagnosticated as incipient or primary tuberculosis.

The diagnosis was founded on about the following assemblage of symptoms: A more or less rapid loss of flesh and strength. Impaired appetite and digestion. Some shortness of breath on exercise, and perhaps slight pains in the chest, referable more particularly to the infraclavicular and interscapular regions of one side. Generally slight hacking cough, and expectoration, if any, slight and of an ordinary catarrhal mucous character. Chest, on inspiration, generally rather flat and narrow, and spare of flesh. Rather harsh bronchial respiration, prolonged expiration, and some degree of bronchophony apparent in the superior portion of one lung on auscultation. More or less marked dullness on percussion on same side.

In this assemblage all, or a majority of the symptoms were apparent in all the cases of this group.

The inhalation of compressed air was combined, in some of these cases, with the exhalation into rarefied air. The same tonic constitutional treatment was also pursued. The results of treatment in this group of cases were evidently influenced very materially by the course and origin of the disease.

A person of naturally healthy constitution, and giving no family history of tuberculosis, but following a sedentary, confined or unhealthy occupation, is attacked with a cold or ordinary catarrhal bronchitis.

Perhaps he has been previously subject, more or less frequently, to similar attacks, but has never found them to coninue so long, or to affect his general flesh and strength as this attack has done. Stooping over his desk or work-bench, or at the sewing machine, as the case may be, there has been a deficient expansion of the lungs, and consequently deficient aeration of the blood. The waste, effete material accumulates in the blood and system, and impairs the general tone and vitality.

Portions of the lungs not fully and freely expanded, under these circumstances, become the seat of chronic congestion, and finally of exudation and infiltration. This process may go on slowly and imperceptibly for a considerable period of time, until finally a slight catarrhal inflammation is the means of starting it into activity.

If allowed to progress unchecked, these cases of phthisis of inflammatory origin, if we so designate them, or of chronic catarrhal pneumonia, according to the German pathologists, follow exactly the same course, to the same fatal termination, as tuberculosis of hereditary origin.

These, however, as has long been recognized, are manageable and curable cases, if taken in the early stage. Change of occupation and out-door life, with free, full, deep breathing and expansion of the lungs, will arrest the progress and cause the re-absorption of such granular or tuberculous deposits in a majority of instances.

If the air-passages are very sensitive and the seat of chronic catarrhal irritation, a change of climate may be necessary to overcome this element in the case.

The first and most important step in the treatment is, however, to establish and fix the habit of full, deep respiration.

Deposits and exudations can only be re-absorbed through the re-establishment of the active capillary circulation in the part, and this is of course dependent on its free expansion. In the accomplishment of this purpose, we believe the inspiration of compressed, and the expiration into rarified air, as already explained, to be the most certain and efficient aid.

Prompt and rapid improvement has taken place in every case of simple inflammatory phthisis that I have placed upon this course of treatment.

In cases involving a tubercular, scrofulous or syphilitic family history, the results are very much more uncertain and unsatisfactory, as regards anything more than temporary relief or improvement.

In tuberculosis, advanced to the stage of softening, I do not think any benefit could be expected from the use of compressed or rarefied air, but, on the contrary, I should fear the occurrence of hæmorrhage from its use, as sometimes happens when patients, so far advanced in tuberculosis, go into too rarefied a mountain atmosphere.

In a number of cases of chronic bronchitis, the use of the compressed and rarefied air has relieved the immediate symptoms of cough and dispnœa very promptly. Whether its continued and persevering use would finally overcome the sensitiveness and chronic congestion of the mucous lining of the air-passages, so as to effect a permanent cure, remains yet to be proved, as regards my own experience. The German writers upon the subject claim that it does so effect a permanent cure, and theoretically its effects upon the capillary circulation of these mucous membranes ought to diminish chronic congestion and thickening.

For emphysema the expiration into rarefied air, is also claimed to be a means of radical cure. In this class of cases, or in valvular diseases of the heart, I have not as yet had an opportunity of testing satisfactorily its effects.

## Figures and Tables

**Figure f1:**